# STRENGTH IN AGE: HARNESSING THE POWER OF THE NATIONAL INSTITUTE ON AGING FOR EARLY-CAREER SCIENTISTS

**DOI:** 10.1093/geroni/igz038.1506

**Published:** 2019-11-08

**Authors:** Marie A Bernard

**Affiliations:** National Institute on Aging, National Institutes of Health, Bethesda, Maryland, United States

## Abstract

The National Institute on Aging (NIA) at the National Institutes of Health, Department of Health and Human Services, supports biomedical and behavioral research with a life-span focus. There is attention to understanding basic processes of aging, improving prevention and treatment of diseases and conditions common in later years, improving the health of older persons, as well as a focus on Alzheimer’s disease and related dementias. The NIA also supports the training and career development of scientists focusing on aging research and the development of research resources. The symposium, meant for junior faculty and emerging scholars, will provide an update on the latest research findings from the NIA followed by a brief update on funding mechanisms. An opportunity is provided to meet and consult with NIA extramural staff.

